# Does point-of-care ultrasonography cause discomfort in patients admitted with respiratory symptoms?

**DOI:** 10.1186/s13049-015-0127-x

**Published:** 2015-06-13

**Authors:** Christian B. Laursen, Erik Sloth, Annmarie Touborg Lassen, Jesper Rømhild Davidsen, Jess Lambrechtsen, Daniel Pilsgaard Henriksen, Poul Henning Madsen, Finn Rasmussen

**Affiliations:** Department of Respiratory Medicine, Odense University Hospital, Sdr. Boulevard 29, 5000 Odense C, Denmark; Institute of Clinical Research, University of Southern Denmark, Odense, Denmark; Department of Anesthesia and Intensive Care, Aarhus University Hospital, Skejby, Denmark; Department of Emergency Medicine, Odense University Hospital, Odense, Denmark; Department of Medicine, Odense University Hospital, Svendborg, Denmark; Department of Clinical Chemistry and Pharmacology, Odense University Hospital, Odense, Denmark; Department of Medicine, Littlebelt Hospital, Vejle, Denmark; Department of Allergy and Respiratory Medicine, Near East University Hospital, Mersin 10, Nicosia North Cyprus Turkey

## Abstract

**Background:**

This study aimed to assess the patient-rated level of discomfort during point-of-care ultrasonography (POCUS) of the heart, lungs and deep veins in a population of patients admitted to an ED with respiratory symptoms and to what extent the patients would accept being assessed by the use of POCUS if they had to be examined for possible disease.

**Methods:**

A questionnaire-based observational study was conducted in an ED. Inclusion criteria were one or more of the following: respiratory rate > 20/min, oxygen saturation < 95 %, oxygen therapy initiated, dyspnoea, cough or chest pain. Patients were examined by the use of POCUS of the heart, lungs and deep veins. Patient-rated level of discomfort and acceptance were assessed using a standardised questionnaire.

**Results:**

The median duration of the sonographic examinations was 12 min (IQR 11–13, range 9–23). The median patient-rated level of discomfort for all three types of POCUS was 1 (IQR 1–1, range 1–8) on a scale from 1 to 10. All but one patient (99.6 % (95 % CI: 98.9-100 %)), would accept being examined by the use of POCUS as a part of routine ED diagnostics.

**Conclusions:**

The patient-rated level of discomfort during POCUS of the heart, lungs and deep veins is very low and the vast majority of patients would accept being assessed by the use of POCUS if the patients once again had to be examined for possible disease**.**

**Electronic supplementary material:**

The online version of this article (doi:10.1186/s13049-015-0127-x) contains supplementary material, which is available to authorized users.

## Background

Diagnostic ultrasonography is a non-invasive and radiation free procedure which also has the advantage of causing minimal patient discomfort and pain [1]. The part of this statement describing patient discomfort and pain seems so obvious that it, by many, would be considered as being an axiom. This is reflected by the fact that the research assessing the benefits and drawbacks of ultrasonography seen from the patient’s perspective is limited. Both patient satisfaction and discomfort during various guided invasive procedures, transvaginal ultrasound, or stress tests involving the use of ultrasound has been assessed [2–8]. However, little is known of the level of patient discomfort during an examination involving diagnostic ultrasound without invasive or stress related procedures. A diagnostic accuracy study of lung ultrasound for the diagnosis of rib fractures reported problems with inflicting pain in some of the patients participating in the study, the degree of pain were however not systematically measured or assessed in the study [9].

Point-of-care ultrasonography (POCUS) is widely used in emergency departments (ED) and its use is expected to rise further in the future [10–12]. The use of POCUS in an ED has been shown to have a positive impact on patient satisfaction and health care consumption [13–16]. Patients admitted to an ED with respiratory symptoms are already in distress due to the symptoms in themselves, whether POCUS in itself causes or aggravates the patient experienced distress in such a patient population has not been explored [17]. Research is needed in order to assess whether POCUS can cause distress and whether patients are willing to accept POCUS as a standard diagnostic procedure in the ED.

A questionnaire-based observational study was conducted in a population of patients admitted to an ED with respiratory symptoms to assess the patient-rated level of discomfort during POCUS of the heart, lungs and deep veins and to what extent the patients would accept being assessed by the use of POCUS if they had to be examined for possible disease.

## Methods

### Design

The study was conducted as a questionnaire-based observational study with data collection alongside two studies assessing the diagnostic accuracy and impact of POCUS in admitted patients with respiratory symptoms [12, 18].

### Setting, study population and ethics

The study took place at the medical section of the ED at Odense University Hospital, Denmark. Corresponding to the inclusion periods of the simultaneously conducted diagnostic accuracy studies two observational periods between November 2010 and May 2011 and December 2011 to March 2013 were used [12, 18]. The ED has approximately 58,800 visits and 12,000 acute admissions each year, whereas nearly 7700 (64 %) of the admissions are related the medical section of the ED. In Denmark, all acute hospital admissions are in public hospitals and occur either as a direct emergency admission or by referral from a general practitioner. Adult patients admitted with respiratory complaints are all admitted to the medical ED, exceptions being trauma patients and patients triaged directly to the department of cardiology on a suspicion of heart disease. Hemodynamic stable patients suspected of having deep vein thrombosis (DVT) or pulmonary embolism (PE), are seen in the department’s out-patient clinic and not admitted to the ED.

The study was conducted according to the Helsinki Declaration and approved by the Committee on Biomedical Research Ethics for The Region of Southern Denmark (ID S-2010074) and the Danish Data Protection Agency (ID 2010-41-5142). Some of the enrolled patients were part in a randomized clinical trial involving the use of POCUS (registered at http://clinicaltrials.gov (NCT01486394)).

### Participants

All patients admitted to the medical ED were following triage screened for study participation by a study investigator. Patients were included if they fulfilled one or more of the following findings:

- Respiratory rate > 20 breaths per minute
- Oxygen saturation < 95 %
- Oxygen therapy initiated

Or if the patients answered yes to ≥ 1 of the following questions when screened:

- Do you feel or have you felt any dyspnoea prior to the admission?
- Do you experience or have you experienced coughing prior to the admission?
- Do you experience or have you experienced chest pain prior to the admission?

### Exclusion criteria

- Permanent mental disability
- Patient age < 18 years
- The sonographic examinations could not be performed within one hour after the primary assessment

### POCUS

As a part of the diagnostic accuracy studies POCUS of the heart, lungs and deep veins was performed within one hour after the primary clinical assessment. All three types of sonography were performed in all of the included patients. The sonographic examinations were performed according to the following protocols:

*Focus Assessed Transthoracic Echocardiography (FATE):* Performed with a M4S or M5S phased array transducer (1.5 – 4.0 / 1.5-4.6 MHz) (General Electric Company) using the FATE protocol [19].

**Fig. 1:**
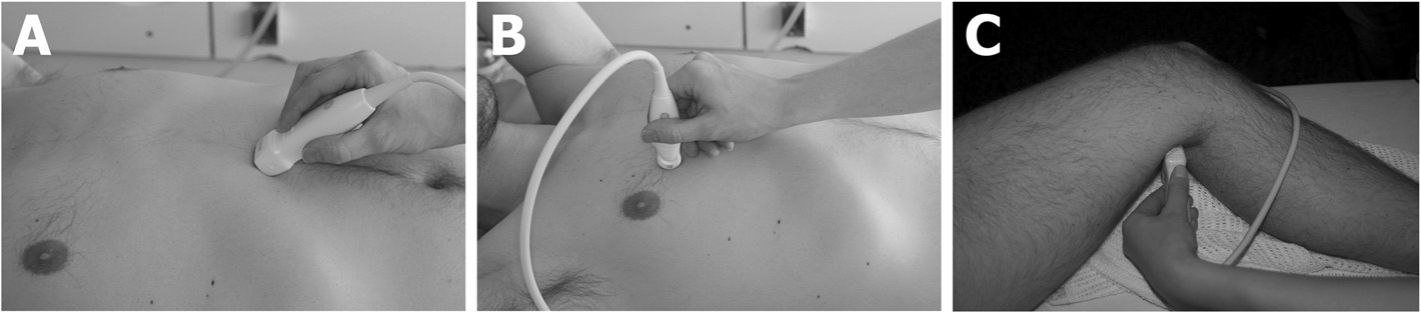
Examples of used transducer positions. **a** Transducer placed for subcostal view during FATE. **b** Transducer placed in an intercostal space at the anterior surface of the chest during FLUS. **c** Transducer placed at the popliteal crease for assessment of the popliteal vein during LCU

*Focused lung ultrasound (FLUS):* Performed with an 8C or 3CRF micro convex transducer (3.5 – 11.5 / 2.0-4.2 MHz) or a C1-5 curved abdominal transducer (2.0-5.0 MHz) (General Electric Company), using a modification of the principles described by Lichtenstein and Volpicelli (Fig. 1) [20, 21].

*Limited compression ultrasonography (LCU):* Performed with a 12 L or ML6-15 linear transducer (5.0 – 13.3 / 4.5-15 MHz) (General Electric Company) according to the American College of Emergency Medicine’s imaging criteria compendium [22].

The patients themselves choose the preferred position to assume while being examined with sonography (e.g. sitting, supine). Examples of transducer positions for the three different sonography protocols are shown in Fig. 1.

All the sonographic examinations were made by the same physician qualified in POCUS (CBL) (FATE examinations > 200, F-LUS examinations > 400, LCU examinations > 200). A Logiq S8 or Vivid S6 (General Electric Company) ultrasound system was used for all examinations.

The time used for the ultrasonographic examinations was defined as the time used from when the transducer touched the patient’s skin for the first time to the examination had been completed and stored on the ultrasound system. The feasibility of the ultrasonographic examinations were assessed according to predefined criteria (see Additional file 1).

### Assessment of patient-rated level of discomfort

To measure the patient-rated level of discomfort during POCUS a simple questionnaire including 4 items was used. Three of the items were Likert items in which the patient were asked to assess the level of discomfort experienced during FATE, FLUS and LCU on a scale from one to ten. The score one corresponded to no discomfort and the score ten corresponded to the worst level of discomfort imaginable by the patient. A score of one to two was believed as being a low score; hence pragmatically it was chosen that if the patient scored any item more than two, the patient was asked whether they could explain the reason for the increased score.

Patient-rated discomfort was chosen in order to encompass a variety of different aspects causing patient discomfort during the examination (e.g. patient position during the examination, pressure being applied on the skin by the transducer, cooperation to the examination while having to cope with respiratory distress).

The final questionnaire item concerned whether the patient would accept or decline being examined by the use of POCUS if the patient once again had to be examined for possible disease. The questionnaire was filled out either by self-completion or by assistance from a study investigator to solely read up the questions. All questionnaires were filled out immediately after the sonographic examination. The questionnaire was solely developed for this study and was not based on any previously validated questionnaires. The questionnaire can be found in its original form (Danish) and an English version in the Additional file 1.

#### Statistical analysis

From the two observation periods data was joined as one cross-section and descriptive statistics were performed including: demographic characteristics, medical history, patient symptoms and vital signs measured at arrival. Time spent for the sonographic examinations were calculated as a median with corresponding 25^th^ and 75^th^ interquartiles (IQR) and range. Feasibility of the sonographic examination was calculated as a proportion with corresponding 95 % confidence interval (CI). Patient-rated levels of discomfort during the three sonographic examinations were calculated as median with corresponding 25^th^ and 75^th^ IQR and range. The number of patients rating the level of discomfort two or lower and the number of patients who would accept being assessed by the use of POCUS if they had to be examined for possible disease were calculated as proportions with corresponding 95 % CIs. Data analysis was conducted using Stata Release Version 11.0 (StataCorp, College Station, TX, USA).

## Results

### Baseline patient characteristics

**Fig. 2:**
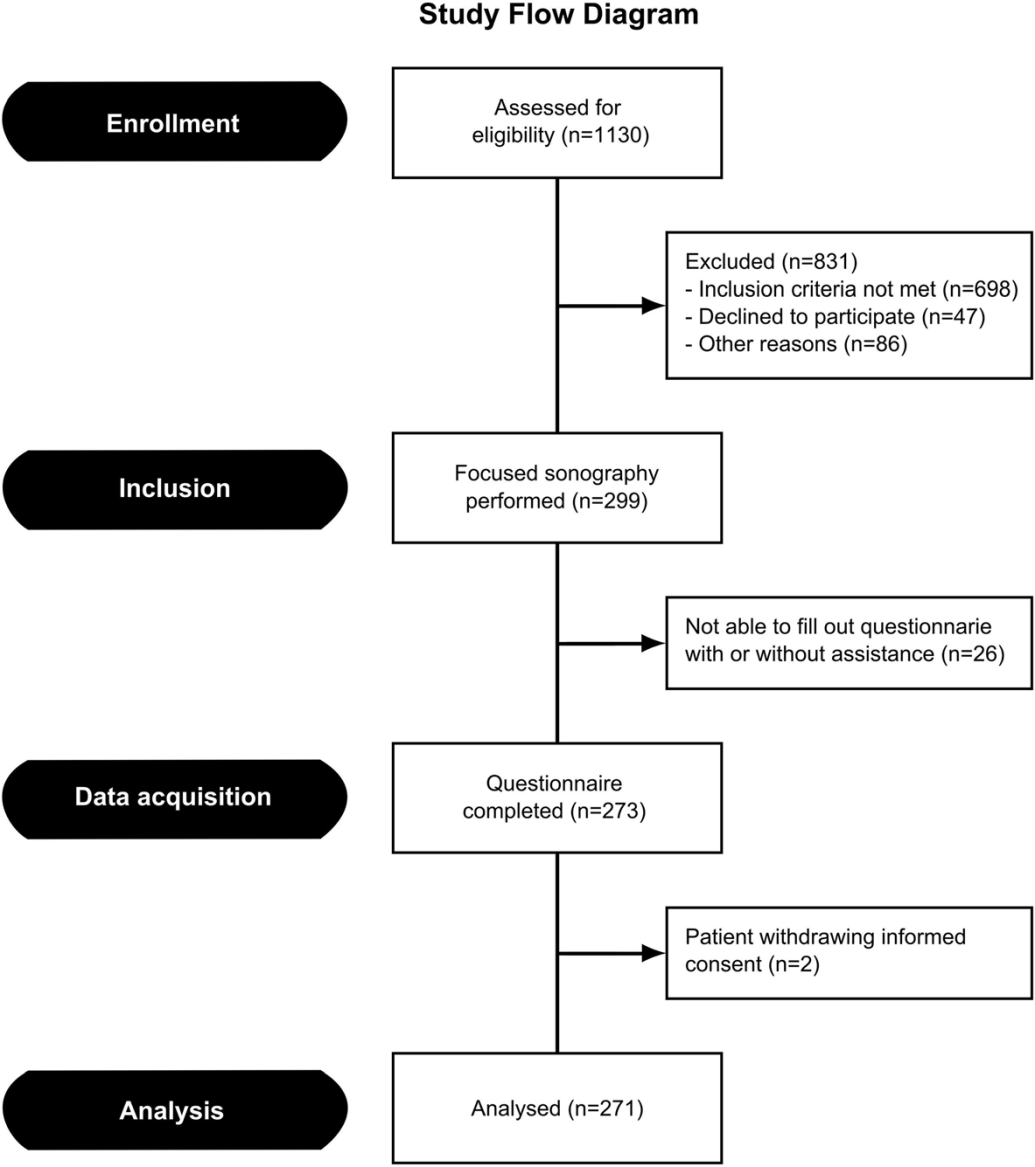
Study flow diagram

A total of 1130 patients were assessed for eligibility. Inclusion criteria were not met in 698 (61.8 %) of the patients and 133 (11.8 %) patients had to be excluded, either due to the patient declining to participate in the study or due to one or more of the exclusion criteria being present. The most common cause for study exclusion was permanent mental disability. A total of 299 (26.5 %) patients remained for study inclusion. Following sonography, 26 patients were not able to fill out the questionnaire with or without assistance and 2 patients withdrew informed consent for study participation during the hospital stay. This left 271 patients remaining in the study. The study flow diagram is presented in Fig. 2 and base-line characteristics of included patients are summarized in Table 1. The results of the screening process for the different patient groups can be found in Table S1 in the Additional file 1.

**Table 1:** Base-line characteristics of the patients

Characteristic	(*N* = 271)
Age – years	
- Median (IQR^a^)	73 (62–81)
- Range	19-100
Sex – no. (%) (95 % CI)	
- Male	103 (38.0) (32.2-43.8)
- Female	168 (62.0) (56.2-67.8)
Smoking status – no. (%) (95 % CI)	
- Never smoked	66 (24.4) (19.2-29.5)
- Current smoker	81 (29.9) (24.4-35.4)
- Previous smoker	112 (41.3) (35.4-47.2)
- No information	12 (4.4) (2.0-6.9)
Medical history – no. (%) (95 % CI)	
- Coronary artery disease	47 (17.3) (12.8-21.9)
- Heart failure	28 (10.3) (6.7-14.0)
- Arterial hypertension	85 (31.4) (25.8-36.9)
- Diabetes mellitus	31 (11.4) (7.6-15.3)
- COPD	110 (40.6) (34.7-46.5)
- Asthma	14 (5.2) (2.5-7.8)
- Other pulmonary disease	15 (5.5) (2.8-8.3)
- Thromboembolic disease	15 (5.5) (2.8-8.3)
- Stroke	24 (8.9) (5.5-12.3)
- Chronic kidney disease	16 (5.9) (3.1-8.7)
- Malignancy	34 (12.6) (8.6-16.5)
- Psychiatric disorder	37 (13.7) (9.5-17.8)
Patient reported symptoms – no. (%) (95 % CI)	
- Subjective experience of dyspnoea	252 (93.0) (89.9-96.0)
- Chest pain	96 (35.4) (29.7-41.2)
- Cough	211 (77.9) (72.9-82.8)
- Sputum production	126 (46.5) (40.5-52.5)
- Purulent sputum	90 (33.2) (27.6-38.9)
- Oedema of one leg	27 (10.0) (6.4-13.6)
- Oedema of both legs	45 (16.6) (12.1-21.1)
- Fever	111 (41.0) (35.1-46.9)
- Weight loss	24 (8.9) (5.5-12.3)
- Weight gain	9 (3.3) (1.2-5.5)
Vital signs at admission	
Respiratory rate – breaths per minute	
- Median (IQR^a^)	20 (16–24)
- Range	10-40
Saturation - %	
- Median (IQR^a^)	96 (93–98)
- Range	75-100
Systolic blood pressure – mmHg	
- Median (IQR^a^)	132 (118–150)
- Range	63-215
Diastolic blood pressure – mmHg	
- Median (IQR^a^)	76 (66–86)
- Range	38-122
Heart rate – beats per minute	
- Median (IQR^a^)	95 (80–110)
- Range	44-169
Temperature - °C	
- Median (IQR^a^)	37.2 (36.6-37.8)
- Range	34.7-40.7

^a^ Interquartile range (IQR) expressed as the 25^th^ and 75^th^

### POCUS

The median duration of the sonographic examinations was 12 min (IQR 11–13, range 9–23). The patient was in sitting position in 186 (62.6 % (95 % CI: 57.1-68.1 %)) and supine position in 111 (37.4 % (95 % CI: 31.8-42.9 %)), respectively. Feasibility for the sonographic examinations were: FATE 99.7 % (95 % CI: 98.1-100 %), FLUS 100 % (95 % CI: 98.8-100 %) and LCU 98.7 % (95 % CI: 96.6-99.6 %).

### Questionnaire items

The median patient-rated level of discomfort for all three types of POCUS was 1 (IQR 1–1, range 1–8). The proportion of patients rating the level of discomfort two or lower was: FATE 96.7 % (95 % CI: 94.5-98.8 %), FLUS 98.2 % (95 % CI: 96.5-99.8 %) and LCU 98.2 % (95 % CI: 96.5-99.8 %). The proportion of patients rating the level of discomfort two or lower for both the heart, lung and deep vein examinations was 95.2 % (95 % CI: 92.6-97.8 %). For patients who rated the level of discomfort higher than two during one or more of the POCUS examinations, the causes are summarized in Table 2. All but one patient (99.6 % (95 % CI: 98.9-100 %)) would accept being examined by the use of POCUS if the patient once again had to be examined for possible disease.

**Table 2:** Patient rated level of discomfort during assessment with POCUS

Type of sonography	(*n* = 271)
Focus Assessed Transthoracic Echocardiography (FATE)	
- Median (IQR^a^)	1 (1–1)
- Range	1-8
Focused Lung Ultrasound (FLUS)	
- Median (IQR^a^)	1 (1–1)
- Range	1-8
Limited compression ultrasonography (LCU)	
- Median (IQR^a^)	1 (1–1)
- Range	1-8

^a^ Interquartile range (IQR) expressed as the 25^th^ and 75^th^

## Discussion

In patients with respiratory symptoms admitted to an ED POCUS of the heart, lungs and deep veins seems to cause little discomfort. The vast majority of these patients would accept being assessed by POCUS if it was a part of the routine ED assessment. The study results provide experimental support for the axiom that claims that ultrasonography is not associated with patient pain or discomfort.

The study population is believed to comprise a representative sample of patients with respiratory symptoms being admitted to an ED in Denmark. Despite the different inclusion criteria and study methods, the base-line characteristics of the patients (Table 1) are comparable with other studies in which ED patients with respiratory symptoms have been assessed [23–28]. As seen in Fig. 2, a relatively large proportion of the patients met inclusion criteria but still had to be excluded. This could have caused selection bias if the excluded patients were sicker than the included patients and thereby possibly more likely to experience discomfort during the examination. Based on the triage colour obtained as a part of the screening process (see Additional file 1, Table 1), the patients in whom POCUS was not performed generally received a lower triage colour than those in which POCUS was performed. The patients who were excluded after POCUS had been performed however generally had a higher triage colour than those patients remaining for study analysis. The potential selection bias introduced by excluding patients after the POCUS could however not be avoided since data could only be collected if the patients were able to fill out the questionnaire with or without assistance.

Other studies have documented the positive impact on patient satisfaction and health care consumption when POCUS is used in an ED [13–16]. That patients generally have a positive attitude towards POCUS is further supported by the study results showing that nearly all included patients would accept being examined using POCUS if it was a standard diagnostic procedure. This seems also to apply for patients experiencing an increased level of discomfort during the examination.

Thirteen patients rated the level of discomfort during one or more of the POCUS examinations higher than two (Table 3). An increased level of discomfort was most often due to an underlying disease or the result of an intervention (e.g. surgery, resuscitation) causing localised pain. Three of the patients with a slight increase in the level of discomfort did not have a specific cause. One patient rated the level of discomfort very high for all three types of POCUS (Table 3, patient no. 143). The patient described a very unpleasant, buzzing sensation going through the entire body, as soon as the transducer touched the patient’s skin. This 44-year old male patient suffered from a known psychiatric disorder, and was admitted to the ED with dyspnoea, chest pain and a cough following inhalation of toxic fumes. No obvious explanation for the patients experienced sensation during POCUS could be given. The same patient would decline being examined by the use of POCUS if the patient once again had to be examined for possible disease.

**Table 3:** Patients rating level of discomfort during assessment with POCUS higher than two in any of the three types of sonography

Patient no.	FATE rating	FLUS rating	LCU rating	Cause for rating > 2
4	3	2	1	Intercostal muscle myalgia
102	3	3	3	No specific cause
139	1	4	1	Rib fractures following chest trauma
143	8	8	8	When the transducer touched the patients skin, the patient described a buzzing sensation going through the entire body
159	1	1	3	Recent pelvic operation with subsequent pain in the femoral region
218	3	2	1	Liver affection with pain in the upper abdomen and intercostal muscle myalgia
234	3	1	1	Tenderness in the epigastric region
259	3	3	3	No specific cause
262	1	3	1	Intercostal muscle myalgia
305	3	2	2	No specific cause
335	4	1	1	Intercostal muscle myalgia
426	8	1	1	Patient had cardiac arrest with subsequent resuscitation prior to examination
428	1	1	3	Dysesthesia in the legs due to neuropathy

The results support the finding of another study, which indicated that underlying disease may cause increased patient discomfort or pain during an ultrasound examination [9]. The number of patients declining to participate in the study comprised 4.2 % of all patients assessed for eligibility and would have comprised 13.6 % of the study population if they all had accepted to participate in study (Fig. 2).

### Limitations

The study was conducted as a single centre study; hence the results can not necessarily be generalized to other EDs or other settings. Additionally, all sonographic examinations were performed by the same physician also affecting the external validity. The advantage of this was, however, a consistent ultrasound examination procedure giving rise to almost elimination of inter-observer-variability. The observed feasibility was very high, which is an indicative of both sufficient pressure being applied and sufficient time being spent performing the examinations. Hence it seems that sonographic examination quality was not compromised in order to decrease possible patient discomfort.

To our knowledge there is no validated questionnaire for the assessment of patient discomfort during sonographic examination; a simple questionnaire developed for the study was used instead. The questionnaire fulfilled face validity. However, a more extensive validation, e.g. content validity was not performed. No data on patient discomfort were available prior to the study, on which an optimal discomfort scale of the questionnaire could be based. It was decided that the primary interest was to identify patients with significant discomfort during the procedure rather than being able to discriminate between patients with a slightly increased level of discomfort. Hence, we developed a non-validated 10-point Likert scale from 1–10 in which 10 corresponded to the worst level of discomfort imaginable by the patient. The scale should thus be able to identify patients with a significantly increased discomfort during the procedure, but would be less useful for discriminating between patients with only a slightly increased level of discomfort.

A potential disadvantage is healthy volunteer bias, i.e. the possibility of selecting patients with certain characteristics among those accepting be examined by ultrasonography. Never the less, as only a minimum of the patients declined the examination being supplemented with POCUS; we have no reason to believe that this type of selection bias may have been a major drawback.

As the questionnaire was performed in the frame of an emergency admission, this could have let to a framing effect resulting in a more positive attitude towards sonographic examination. The frame however reflects the setting and patients in which POCUS is used in clinical practice. If the patients had been asked to fill out the questionnaire later on, the time given for patient reflection, the presence of framing, these potential effect modifiers could affected the study results negatively due to primarily recall bias.

### Clinical implications

Patients admitted with respiratory symptoms may experience stress and anxiety due to both symptoms caused by the underlying disease and simply by being admitted to hospital. Despite of this, POCUS is generally well accepted and in itself causes little discomfort in these patients. Patients with comorbidity causing localised pain (e.g. intercostal muscle myalgia, rib fractures, neuropathy) may experience an increased level of discomfort. When performing POCUS in patients with such conditions, the patient should prior to the examination be informed of an increased risk of experiencing discomfort. Still, POCUS should not be withheld in these patients since the patient-rated level of discomfort is low and the patients despite of the increases discomfort nonetheless accepts being examined by the use of POCUS.

## Conclusion

In patients admitted to an ED with respiratory symptoms, the patient-rated level of discomfort during POCUS of the heart, lungs and deep veins is very low. The vast majority of patients would accept being examined by the use of POCUS if the patients once again had to be examined for possible disease**.**

## Additional file

Additional file 1:
**Feasibility of the sonographic examinations.**
