# Supplementary material for: Does point-of-care ultrasonography cause discomfort in patients admitted with respiratory symptoms?
Source: Scand J Trauma Resusc Emerg Med. 2015 Jun 13;23:46. doi: 10.1186/s13049-015-0127-x (PMC4465167; doi:10.1186/s13049-015-0127-x)
Supplement: Additional file 1: — Feasibility of the sonographic examinations. [file 13049_2015_127_MOESM1_ESM.doc]

**Additional file 1**

**Feasibility of the sonographic examinations**

**Focused echocardiography:**

Defined as the percentage of patients in which it was possible to:

- determine whether there was a pericardial effusion present or not
- determine left ventricle ejection fraction
- determine whether marked dilatation of the right ventricle was present or not

**Lung ultrasound:**

Defined as the percentage of patients in which it was possible to perform focused sonographic examination of the anterior, lateral and posterior surface of the chest.

**Limited compression ultrasonography:**

Defined as the percentage of patients in which, using sonography, it was possible to visualise the common femoral, the superficial femoral and the popliteal veins in both legs.

**Table 1. Screening characteristics of the patients assessed for eligibility**

| **Characteristic** | **All screened patients**  **(n=1130)** | **Excluded patients**  **(n = 831)** | **US performed but excluded from study analysis***  **(n = 28)** | **US performed and included in study analysis**  **(n = 271)** |
| --- | --- | --- | --- | --- |
| Age – years |  |  |  |  |
| - Median(IQR^a^) - Range | 71 (54-81)  1-101 | 69 (49-81)  1-101 | 76 (64-86)  41-98 | 73 (61-81)  19-100 |
| Sex – no. (%) |  |  |  |  |
| - Male - Female | 511 (45.2)  619 (54.8) | 392 (47.2)  439 (52.8) | 16 (57.1)  12 (42.9) | 103 (38.0)  168 (62.0) |
| Triage colour^b^ – no. (%) |  |  |  |  |
| - Green - Yellow - Orange - Red - No colour registered | 410 (36.3)  446 (39.5)  234 (20.7)  37 (3.3)  3 (0.2) | 336 (40.4)  323 (38.9)  144 (17.3)  26 (3.1)  2 (0.2) | 1 (3.6)  8 (28.6)  15 (53.6)  4 (14.3)  - | 73 (27.0)  115 (42.6)  75 (27.8)  7 (2.6)  1 (0.004) |
| Inclusion criteria – no. (%) |  |  |  |  |
| - Respiratory rate > 20 b/min - Saturation < 95% - Treatment with oxygen - Dyspnoea - Cough - Chest pain | 244 (21.6)  334 (29.6)  350 (31.0)  480 (42.5)  388 (34.3)  183 (16.2) | 112 (13.5)  156 (18.8)  175 (21.1)  218 (26.2)  170 (20.5)  84 (10.1) | 22 (78.6)  25 (89.3)  25 (89.3)  10 (35.7)  7 (25.0)  3 (10.7) | 110 (40.6)  153 (56.5)  150 (55.4)  252 (93.0)  211 (77.9)  96 (35.4) |
| Exclusion criteria – no. (%) |  |  |  |  |
| - Permanent mental disability - Patient age < 18 years - US not be performed | 64 (5.7)  20 (1.8)  24 (2.1) | 64 (7.7)  20 (2.41)  184 (22.1) | -  -  - | -  -  - |

^*^ Excluded either due patient not being able to fill out questionnaire with or without assistance or due to patient withdrawing informed consent.

^a^ Interquartile range (IQR) expressed as the 25^th^ and 75^th^

^b^ Triage colour were assigned according to a modified version of Adaptiv Processtriage ( Lethvall S. ADAPT - Adaptiv Processtriage/VITALHISTORIER, version 1.1.2008. Giltiga 080424-090531 (Sweden))

**Questionnaire used for the study**

**Original questionnaire used in the study (Danish)**

1. På en skala fra 1 til 10, hvor 1 er intet ubehag og 10 er den værst tænkelige form for ubehag du kan forestille dig, hvor ubehagelig var hjerteskanningen?
2. På en skala fra 1 til 10, hvor 1 er intet ubehag og 10 er den værst tænkelige form for ubehag du kan forestille dig, hvor ubehagelig var lungeskanningen?
3. På en skala fra 1 til 10, hvor 1 er intet ubehag og 10 er den værst tænkelige form for ubehag du kan forestille dig, hvor ubehagelig var skanningen af blodkarrene i benene?
4. Hvis du atter i forbindelse med undersøgelse for sygdom, fik tilbudt at blive undersøgt med ultralydsscanningerne, vil du da gerne have scanningerne foretaget eller gerne have dig frabedt at få scanningerne foretaget?

**Translated version of the questionnaire used in the study**

1. On a scale from 1 to 10, in which the score one corresponds to no discomfort and the score ten corresponds to the worst level of discomfort imaginable to you, how would you grade the level of discomfort experienced during the sonographic examination of the heart?
2. On a scale from 1 to 10, in which the score one corresponds to no discomfort and the score ten corresponds to the worst level of discomfort imaginable to you, how would you grade the level of discomfort experienced during the sonographic examination of the lungs?
3. On a scale from 1 to 10, in which the score one corresponds to no discomfort and the score ten corresponds to the worst level of discomfort imaginable to you, how would you grade the level of discomfort experienced during the sonographic examination of the deep veins in the legs?
4. Would you accept or decline being assessed by the use of these ultrasound examinations if you once again had to be examined for possible disease?
